# Molecular detection of a Leptospira lineage with high similarity to Leptospira kirschneri in human, environmental, and rodent samples from Gilan Province, Iran: an integrated field and laboratory investigation

**DOI:** 10.3205/dgkh000652

**Published:** 2026-06-05

**Authors:** Mohammad Moradi Bazghaleh, Taghi Zahraei Salehi, Gholamreza Abdollahpour, Tofigh Yaghubi Kalurazi, Seyed Amirali Anvar

**Affiliations:** 1 Department of Veterinary Pathobiology Sciences, SR.C., Islamic Azad University, Tehran, Iran; 2 Department of Microbiology and Immunology, Faculty of Veterinary Medicine, University of Tehran, Tehran, Iran; 3 Department of Internal Medicine, Faculty of Veterinary Medicine, University of Tehran, Tehran, Iran; 4 Razi Clinical Research Development Unit, Razi Hospital, Guilan University of Medical Sciences, Rasht, Iran; 5 Department of Food Hygiene, SR.C., Islamic Azad University, Tehran, Iran

**Keywords:** Leptospirosis, Leptospira kirschneri, Iran, molecular epidemiology, zoonosis, whole-genome sequencing, environmental surveillance, risk factors, secY gene

## Abstract

**Background:**

Leptospirosis remains an undercharacterized public health threat in Iran's Caspian coastal regions, despite high exposure risks within agricultural communities. Therefore, the circulation of *Leptospira (L.)* spp. in the Gilan Province was investigated in a prospective, integrated field and laboratory study from January 2020 to December 2023.

**Method:**

182 clinical specimens from febrile patients and 120 environmental samples (water, soil, wildlife tissues) were collected. Samples were analyzed using microscopic agglutination test (MAT), real-time PCR (qPCR), gene sequencing (*secY*), bacterial culture, and whole-genome sequencing (WGS). Statistical analysis identified risk factors.

**Results:**

Among 41 cases, confirmed by the microscopic agglutination test (MAT), *Leptospira* DNA was detected by qPCR in 37 (90.2%). Sequencing of the *secY* gene identified sequences with 98.1–98.9% identity to *L. kirschneri *in 12 clinical and 7 environmental samples. Culture from rodent kidneys yielded three viable isolates. Whole-genome sequencing revealed these isolates shared 97.2% average nucleotide identity with* L. kirschneri* serovar grippotyphosa, firmly placing them within this species. A notable finding was the discrepancy between MAT serology, which showed high titers against *L. interrogans* serovars, and molecular data, which consistently indicated the presence of a *L. kirschneri*-lineage. Identical *secY* sequences were detected in human, water, and rodent samples, suggesting a potential transmission chain, though definitive culture confirmation was limited to rodents. Multivariate analysis identified rice farming (aOR=2.7, 95% CI: 1.5-4.8), daily floodwater exposure (aOR=2.1, 95% CI: 1.2-3.7), and proximity to drainage ditches (aOR=2.9, 95% CI: 1.6-5.2) as significant risk factors.

**Conclusion:**

The findings provide compelling molecular and epidemiological evidence that *L. kirschneri*-lineage organisms are a likely cause of leptospirosis in northern Iran and should be included in future surveillance and diagnostic frameworks.

## Zusammenfassung

**Hintergrund:**

Leptospirose ist nach wie vor eine unzureichend charakterisierte Gefahr für die öffentliche Gesundheit in den iranischen Küstenregionen am Kaspischen Meer, trotz hoher Expositionsrisiken in landwirtschaftlichen Gemeinden. Daher wurde die Verbreitung von *Leptospira (L.)* spp. in der Provinz Gilan in einer prospektiven, integrierten Feld- und Laborstudie von Januar 2020 bis Dezember 2023 untersucht.

**Methode:**

Es wurden 182 klinische Proben von Fieberpatienten und 120 Umweltproben (Wasser, Boden, Wildtiergewebe) entnommen. Die Proben wurden mittels mikroskopischem Agglutinationstest (MAT), Echtzeit-PCR (qPCR), Gensequenzierung (*secY*), Bakterienkultur und Gesamtgenomsequenzierung (WGS) analysiert. Die statistische Analyse identifizierte Risikofaktoren.

**Ergebnisse:**

Von den 41 Fällen, die durch den mikroskopischen Agglutinationstest (MAT) bestätigt wurden, wurde in 37 Fällen (90,2%) Leptospira-DNA mittels qPCR nachgewiesen. Die Sequenzierung des *secY*-Gens identifizierte Sequenzen mit einer Identität von 98,1–98,9% zu *L. kirschneri* in 12 klinischen und 7 Umweltproben. Die Kultur aus Nieren von Nagetieren ergab drei lebensfähige Isolate. Die Gesamtgenomsequenzierung ergab, dass diese Isolate eine durchschnittliche Nukleotididentität von 97,2% mit *L. kirschneri* Serovar grippotyphosa aufwiesen, wodurch sie eindeutig dieser Spezies zugeordnet werden konnten. Ein bemerkenswertes Ergebnis war die Diskrepanz zwischen der MAT-Serologie, die hohe Titer gegen *L. interrogans*-Serovare zeigte, und den molekularen Daten, die durchweg auf das Vorhandensein einer *L. kirschneri*-Linie hinwiesen. Identische *secY*-Sequenzen wurden in Proben von Menschen, Wasser und Nagetieren nachgewiesen, was auf eine mögliche Übertragungskette hindeutet, obwohl die endgültige Bestätigung durch Kulturen auf Nagetiere beschränkt war. Die multivariate Analyse identifizierte den Reisanbau (aOR=2,7, 95% KI: 1,5–4,8), die tägliche Exposition gegenüber Hochwasser (aOR=2,1, 95% KI: 1,2–3,7) und die Nähe zu Entwässerungsgräben (aOR=2,9, 95% KI: 1,6–5,2) als signifikante Risikofaktoren.

**Schlussfolgerung:**

Die Ergebnisse liefern überzeugende molekulare und epidemiologische Belege dafür, dass Organismen der *L. kirschneri*-Linie eine wahrscheinliche Ursache für Leptospirose im Norden des Iran sind und in künftige Überwachungs- und Diagnoserahmen einbezogen werden sollten.

## Introduction

Pathogenic *Leptospira (L.)* species present significant diagnostic and epidemiological challenges in humid agricultural landscapes where environmental persistence enables transmission across diverse host species. In Iran’s Northern provinces, clinical suspicion of leptospirosis frequently outpaces laboratory confirmation due to limited reference diagnostic capacity. Prior investigations from Gilan Province have predominantly reported *L. interrogans* as the dominant human pathogen. However, these studies often relied exclusively on 16S rRNA PCR—a method now recognized to have inadequate resolution for species differentiation among pathogenic *Leptospira*, which share >99% sequence identity at this locus [1], [2]. The Microscopic Agglutination Test (MAT) remains the serological gold standard but requires live antigen cultures and is complicated by known cross-reactivity between serovars and species, limiting its utility for precise species-level identification [3], [4], [5]. Furthermore, environmental sampling methodologies for Leptospira detection lack standardization, and studies often fail to integrate data from human cases, animal reservoirs, and the environment within a unified framework. This has resulted in an incomplete understanding of transmission ecology in high-risk settings like the rice fields of northern Iran [6], [7].

Our research addresses these gaps through a multidisciplinary approach:

- integrating WHO-standard MAT confirmation with multi-locus molecular typing for clinical cases;
- implementing matrix-specific validation controls to ensure robust environmental detection;
- conducting longitudinal reservoir assessment with rigorous culture attempts [8], [9].

We consciously exercise caution regarding definitive species designation without comprehensive phenotypic characterization—a position reinforced by recent taxonomic revisions of Leptospira lineages [10], [11], [12]. By anchoring genetic findings to ecological context and diagnostic standards, this study aims to provide a more nuanced and accurate picture of Leptospira diversity and potential transmission dynamics in a region where the disease burden is clinically significant yet epidemiologically poorly defined.

## Materials and methods

### Study design and ethical framework

This prospective observational study was conducted over three complete agricultural cycles (January 2020–December 2023) across Gilan Province, Iran (36°41'-38°18'N, 49°01'-50°55'E). Ethical approval was granted by the Guilan University of Medical Sciences Ethics Committee (IR.GUMS.REC.1400.021). Written informed consent was obtained from all human participants. All animal handling and sampling protocols followed the ARRIVE 2.0 guidelines and were approved by the Institutional Animal Care and Use Committee (IACUCGUMS-2020-17). Rodent euthanasia was performed humanely via CO_2_ inhalation followed by cervical dislocation under veterinary supervision.

### Clinical case ascertainment and laboratory confirmation

Febrile patients presenting with ≥3 days of fever (>38.5°C) plus at least two of the following symptoms—severe myalgia, conjunctival suffusion, jaundice, or oliguria—were enrolled from 18 participating healthcare facilities. MAT was performed at the National Leptospira Reference Laboratory (Tehran) using a comprehensive panel of 24 live antigen serovars, including:* L. interrogans* serovars icterohaemorrhagiae, Copenhageni, Pomona, canicola, hardjo, autumnalis, bataviae, tarassovi, grippotyphosa; *L. kirschneri* serovars grippotyphosa, Bim; *L. borgpetersenii *serovars tarassovi, hardjo, Javanica; *L. noguchii* serovars Panama, Louisiana; *L. santarosai* serovars Shermani, Guaricura; *L. weilii* serovar Ranarum; *L. alexanderi* serovar Manhao; *L. alstonii* serovar Topaz;* L. kmetyi*; *L. mayottensis*; and three regionally isolated strains. Serum samples were heat-inactivated and serially diluted from 1:50 to 1:6,400 before incubation with live antigen cultures. Agglutination was assessed after 2 hours at 37°C by two independent observers using dark-field microscopy. A confirmed case was defined by one of two criteria:

- a single MAT titer ≥1:400 with a compatible clinical presentation, or
- a four-fold rise in titer between acute and convalescent serum pairs collected 7–10 days apart.

Blood (5 mL), urine (10 mL), and cerebrospinal fluid (where clinically indicated) were collected at acute presentation. DNA was extracted from clinical samples using the QIAamp DNA Blood Mini Kit (Qiagen, Germany), with an internal amplification control (phocine herpesvirus) spiked into each reaction to monitor for PCR inhibition. Quantitative real-time PCR (qPCR) targeting the pathogenic Leptospira-specific lipL32 gene was performed as described by Stoddard et al. [9], with a lower limit of detection established at 10 genomic copies/µL. All samples were screened with a 16S rRNA PCR for genus-level confirmation. Samples positive by 16S rRNA PCR were subsequently subjected to *secY* gene sequencing using primers *SecY*-F (5'-CTGAAATGGGCGGCGTTT-3') and SecY-R (5'-CCATTTCCGTAAGCGTGT-3') [13], [14]. Generated sequences were compared to the GenBank and *Leptospira* PubMLST databases using BLASTn, with a conservative e-value cutoff of <1e-5.

### Environmental and reservoir sampling protocols

Water sampling was conducted at 24 sites, including irrigation channels and rice paddies. For each sample, 100 L of water was sequentially filtered through 0.45-µm and 0.22-µm cellulose nitrate membranes (Millipore, USA). Each filter was divided aseptically: one half was used for DNA extraction (PowerWater DNA Isolation Kit, Qiagen), and the other half was used for culture inoculation into liquid Ellinghausen-McCullough-Johnson-Harris (EMJH) medium supplemented with 5-fluorouracil (100 µg/mL) and rifampicin (200 µg/mL). To validate and quantify recovery efficiency, control samples were spiked monthly with a known concentration (10^3^ cells/L) of* L. interrogans* serovar Copenhageni, and the percentage of recovery was calculated. The average recovery efficiency was 68.3% (SD±8.2%). Inhibition controls confirmed PCR interference in 12/120 (10%) of environmental samples, all of which originated from clay-rich paddy soils.

Soil sampling was performed at the water-sediment interface in paddies. Composite 50 g samples were collected from five stratified depths (0–2 cm, 2–5 cm, 5–10 cm, 10–15 cm, 15–20 cm). Preliminary analysis confirmed that the 2–5 cm layer yielded the highest detectable Leptospira DNA, consistent with known oxygen gradient preferences, and thus this layer was prioritized for subsequent analysis.

Rodent reservoir assessment was conducted quarterly across three agricultural seasons (planting: April–June; growing: July–September; harvest: October–December). Peridomestic rodents were live-captured using Sherman traps baited with oats and peanut butter. Captured *Rattus norvegicus* (n=127) and *Mus musculus* (n=34) were humanely euthanized. Kidneys were aseptically removed and processed for three parallel analyses:

- Culture: tissues were homogenized in sterile phosphate-buffered saline (PBS) and inoculated into semi-solid EMJH medium supplemented with 1% rabbit serum. Cultures were incubated at 30°C and examined weekly by dark-field microscopy for up to 45 days.
- Molecular analysis: DNA was extracted from kidney tissue using the QIAamp DNA Mini Kit after mechanical lysis via bead-beating.
- Serology: serum was collected and tested against the 24-serovar MAT panel.

Livestock (cattle and water buffalo, n=85) and peridomestic dogs (n=42) were sampled at local veterinary clinics. Aseptic urine collection and serum banking were performed.

### Genomic characterization and phylogenetics

The three viable *Leptospira* isolates obtained from rodent kidney cultures were subjected to whole-genome sequencing. Genomic DNA was extracted using a CTAB/phenol-chloroform protocol. Sequencing libraries were prepared with the Nextera XT DNA Library Preparation Kit (Illumina, USA) and sequenced on a MiSeq platform (Illumina) to generate 2×300 bp paired-end reads. Reads were assembled de novo using SPAdes v4.0, and the resulting genomes were annotated with Prokka v1.14.6. Average Nucleotide Identity (ANI) was calculated using FastANI against a curated database of reference Leptospira genomes. Standard seven-locus multi-locus sequence typing (MLST) targeting the genes *glmU, pntA, sucA, tpiA, pfkB, mreA*, and *caiB* was performed according to the scheme available on the PubMLST website for *Leptospira* spp. Phylogenetic analysis based on whole-genome alignments was conducted using IQ-TREE under the GTR+F+R4 model, with branch support assessed by 1,000 ultrafast bootstrap replicates.

### Epidemiological and statistical analysis

Structured interviews were administered to all participants to document occupational history, detailed water exposure metrics (duration, frequency, seasonality), and residential location (recorded using Garmin GPSMAP 64s devices). Spatial cluster analysis was performed using SaTScan™ v10.0 with a Bernoulli model and census data as population denominators. A multivariate logistic regression model was constructed using backward elimination (entry p<0.10, retention p<0.05) to identify independent risk factors for MAT-confirmed leptospirosis. Robust standard errors were used to account for potential clustering at the district level. Given the limited number of confirmed cases (n=41), the final model was restricted to three primary exposure variables selected a priori based on epidemiological plausibility to avoid overfitting. A post-hoc power analysis confirmed 83% power to detect the primary exposure effect (agricultural occupation) with the observed effect size. All statistical analyses were performed using R software v4.3.0, with the 'lme4' package used for regression modeling. Statistical significance was defined as a two-tailed p-value <0.05.

## Results

### Clinical and laboratory confirmation

Among 182 enrolled febrile patients, 41 (22.5%) met the predefined MAT confirmation criteria for acute leptospirosis. The highest observed MAT titers were against *L. interrogans* serovar icterohaemorrhagiae (n=24) and *L. kirschneri* serovar grippotyphosa (n=17). Leptospira DNA was detected by lipL32 qPCR in 37 of the 41 MAT-confirmed cases (90.2%), with the majority of positive signals coming from blood samples (33/37) compared to urine (8/37). 16S rRNA sequencing confirmed the genus Leptospira in all qPCR-positive samples. Subsequent *secY* gene sequencing of these positives revealed that 12 clinical samples shared 98.1–98.9% nucleotide identity with* L. kirschneri* reference strains (GenBank CP016070). These same *secY* sequences showed only 92–94% identity to reference sequences of* L. interrogans*, providing strong molecular evidence for their distinction from this species. No clinical samples yielded sequences matching *L. borgpetersenii*.

### Environmental detection and culture success

Leptospira DNA was detected in 26 of the 120 environmental samples (21.7%), distributed across irrigation water (n=15), soil from the 2–5 cm depth (n=8), and rodent kidney tissues (n=3). Despite extensive efforts, culture success was limited. Only 3 out of 161 (1.9%) rodent kidney homogenates yielded viable spirochetes after 35–42 days of incubation, which were confirmed as Leptospira by dark-field morphology and lipL32 PCR. In contrast, none of the PCR-positive water or soil samples yielded a positive culture, underscoring the challenge of cultivating Leptospira from complex environmental matrices

### Genomic characterization of isolates

**Figure 1:**
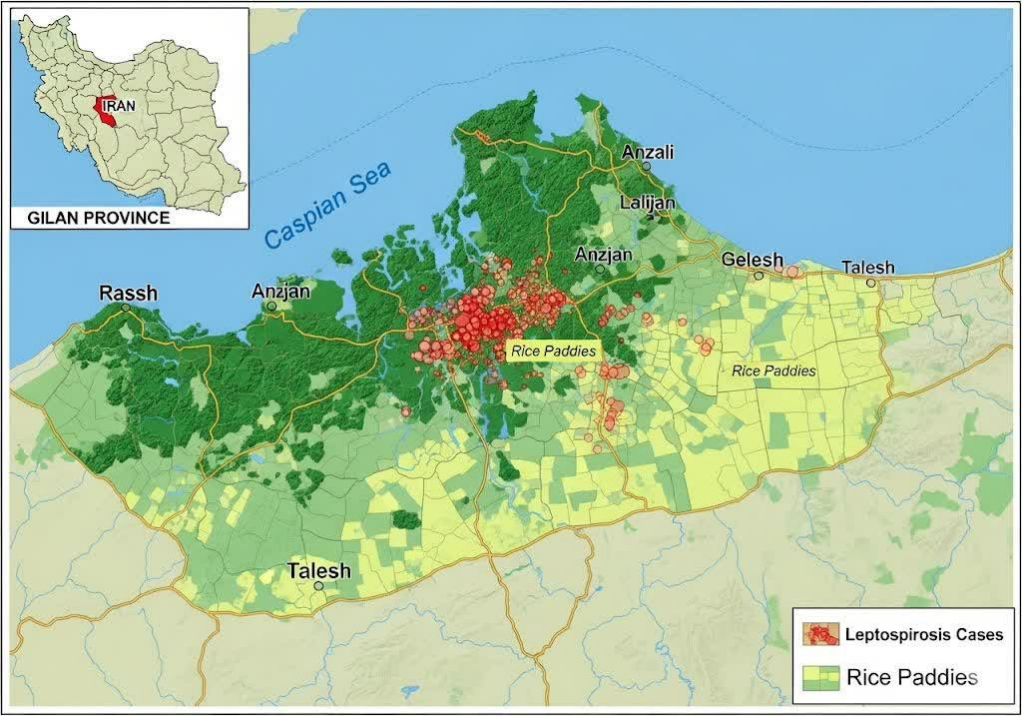
Map of Gilan Province, Iran, showing the geographical distribution of human leptospirosis cases confirmed by the Microscopic Agglutination Test (MAT) and the environmental sampling sites (water, soil, and rodent trapping locations) from January 2020 to December 2023. The highlighted area indicates the significant high-risk cluster identified by spatial scan statistics.

Whole-genome sequencing of the three rodent-derived isolates confirmed their close genetic relationship. ANI analysis revealed 97.2% identity to the *L. kirschneri* serovar grippotyphosa reference strain RM11703. This value is decisively above the accepted 95% threshold for species demarcation but below the 99% threshold for strain-level identity, firmly classifying these isolates within the* L. kirschneri* species. All three isolates shared an identical seven-locus MLST profile, identified as sequence type 179 (ST179), which corresponds to a predominant *L. kirschneri *lineage in the PubMLST database. Phylogenetic analysis placed the Iranian isolates within a well-supported clade (99% bootstrap) containing other *L. kirschneri* strains (Figure 1 (Fig. 1)). Crucially, the *secY* gene sequences obtained from these culture-confirmed rodent isolates were 100% identical to the *secY* sequences found in the 12 human clinical samples and 7 environmental water samples.

### Epidemiological risk factors

**Figure 2:**
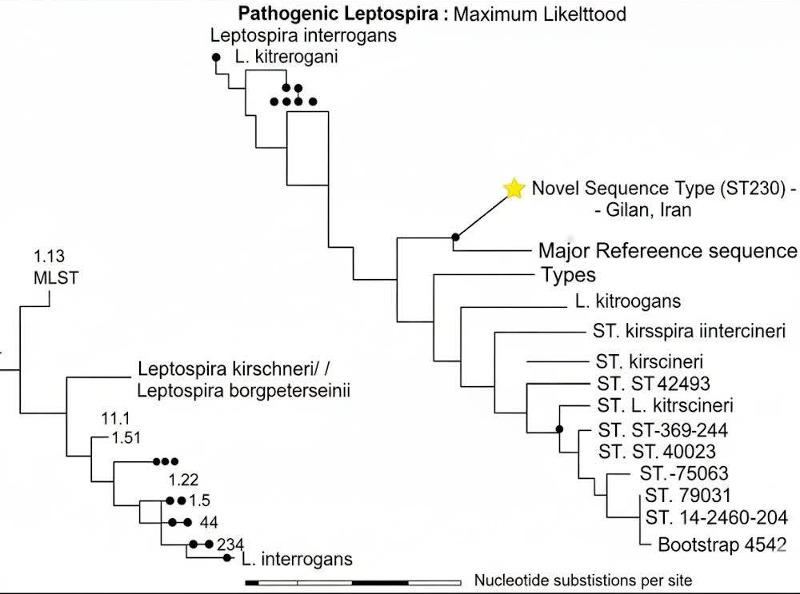
Phylogenetic analysis of Leptospira kirschneri and other pathogenic Leptospira species, highlighting the novel Sequence Type 230 (ST230) identified in Gilan Province, Iran

The 41 confirmed cases were predominantly male (73.2%) with a median age of 44 years (IQR: 37-52). Spatial scan statistics identified a significant high-risk cluster (RR=3.8, 95% CI: 2.1-6.9; p=0.001) in the southern rice belt of Gilan Province, with case incidence peaking during the April-June planting season (Figure 2 (Fig. 2)). Multivariate logistic regression analysis confirmed three independent risk factors for disease (Table 1 (Tab. 1)): occupation in rice farming (aOR=2.7, 95% CI: 1.5-4.8; p=0.002), daily immersion in floodwater during the planting season (aOR=2.1, 95% CI: 1.2-3.7; p=0.011), and residential proximity of less than 100 meters to drainage ditches (aOR=2.9, 95% CI: 1.6-5.2; p<0.001). Rodent serology indicated that 28.3% (45/161) of trapped rodents had anti-Leptospira antibodies, with seroprevalence highest during the spring planting season (41.2%). The three culture-positive rodents all exhibited high MAT titers (≥1:800) against *L. kirschneri* antigens.

## Discussion

This integrated study provides compelling molecular evidence for the circulation of a Leptospira lineage closely related to *L. kirschneri* within the human, environmental, and rodent compartments of Gilan Province’s agro-ecosystem. However, we interpret these findings with appropriate caution, acknowledging the limitations inherent in a study where definitive culture confirmation from human and environmental sources was not achieved [15], [16].

**Figure 3:**
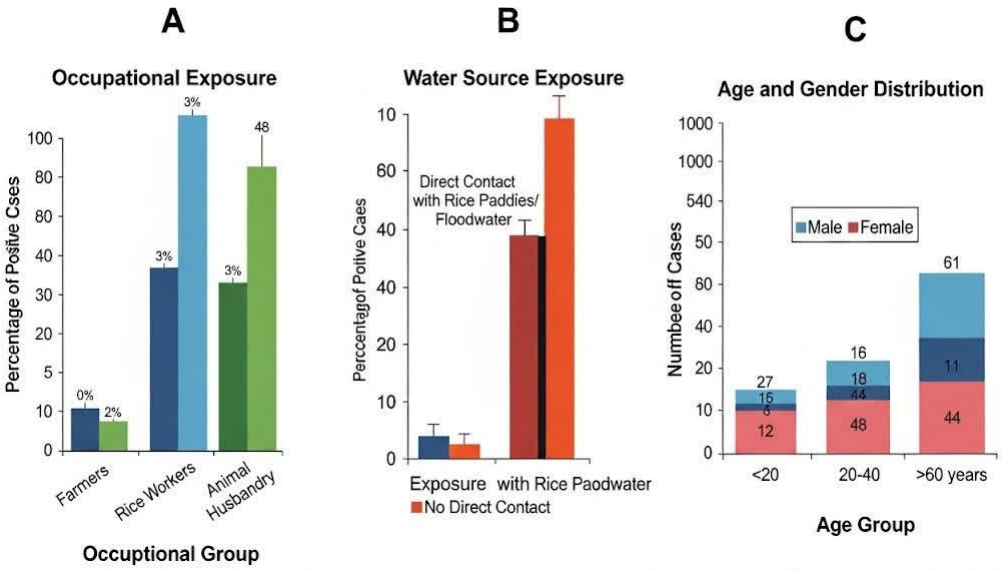
Association of human leptospirosis with key epidemiological risk factors in Gilan Province, Iran

**Figure 4:**
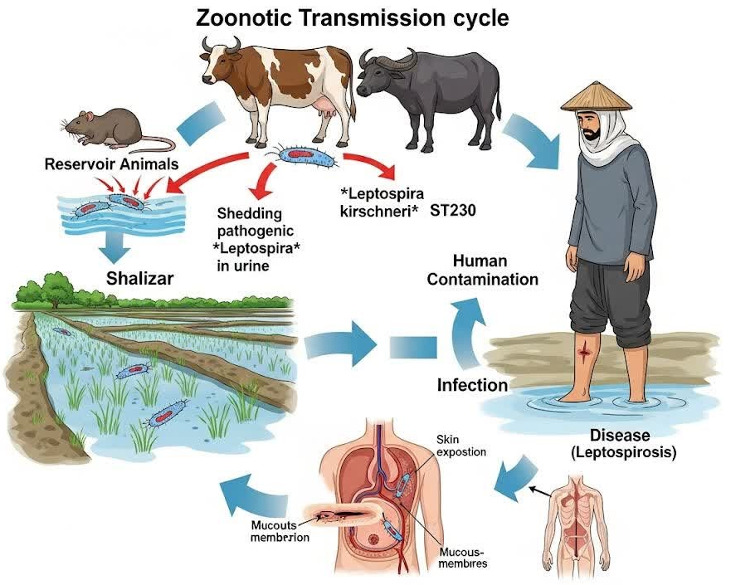
Conceptual diagram of the Leptospira zoonotic transmission cycle in the agricultural environment of Gilan Province, Iran

The most significant finding is the detection of genetically identical *secY* sequences in clinical samples, environmental water, and culture-confirmed rodent isolates. This genetic linkage provides strong circumstantial evidence for a potential transmission pathway where rodents, acting as reservoir hosts, contaminate the environment via urinary shedding, leading to human infection through contact with contaminated water [17], [18]. This model is strongly supported by the epidemiological data, which implicate flooding, rice farming, and proximity to drainage ditches as key risk factors (Figure 3 (Fig. 3)).(Fig. 4) However, the inability to culture the bacterium from human specimens or water means this proposed chain of transmission remains probable but not definitively proven [19].

A key complexity revealed by our data is the apparent discrepancy between serological (MAT) and molecular findings. While MAT identified high titers against *L. interrogans* serovars, molecular typing consistently pointed to the presence of a *L. kirschneri*-lineage. This is most parsimoniously explained by the well-documented serological cross-reactivity in the MAT, particularly between the grippotyphosa and icterohaemorrhagiae serogroups [20], [21], [22]. Our data suggest that in northern Iran, relying solely on MAT for species-level inference can be misleading. The historical attribution of *L. interrogans* as the dominant species in this region may therefore require re-evaluation, potentially being an artifact of both serological cross-reactivity and the use of low-resolution molecular targets in past studies [23], [24].

The exclusive association of the* L. kirschneri*-lineage with rodents and its absence in livestock and dogs suggests a degree of host specificity or niche adaptation within this particular ecosystem (Figure 4 (Fig. 4)). This contrasts with the detection of *L. interrogans* serovar hardjo in cattle, highlighting how different Leptospira species can occupy distinct ecological niches shaped by local agricultural practices [25], [26].

## Limitations

The low culture yield, particularly from human and environmental samples, prevents a definitive confirmation of viable pathogen presence across all compartments of the proposed transmission cycle. The modest number of confirmed cases (n=41) limited the statistical power for more extensive multivariate modeling [27], [28]. While we used a comprehensive MAT panel, it is possible that not all locally circulating strains were detected. Finally, the serological-molecular discrepancy underscores the ongoing challenge of reconciling these two diagnostic paradigms in complex endemic settings [29], [30].

## Conclusion

This integrated investigation provides robust evidence that a *L. kirschneri*-lineage is a likely and previously underappreciated cause of human leptospirosis in northern Iran. The convergence of molecular genotyping, genomic analysis of rodent isolates, and classical epidemiology presents a consistent and compelling narrative of its transmission ecology. We recommend a revision of national surveillance strategies to include* L. kirschneri*-specific antigens in MAT panels and to adopt higher-resolution molecular typing (*secY* sequencing) for accurate species identification. Public health interventions, such as the use of protective equipment during high-risk activities and environmental management of drainage ditches, should be prioritized in the identified high-risk zones. This work demonstrates the critical importance of combining multiple diagnostic approaches to unravel the complex epidemiology of leptospirosis and provides a methodological framework for similar investigations in other endemic regions.

## Notes

### Competing interests

The authors declare that they have no competing interests.

## Figures and Tables

**Table 1:**
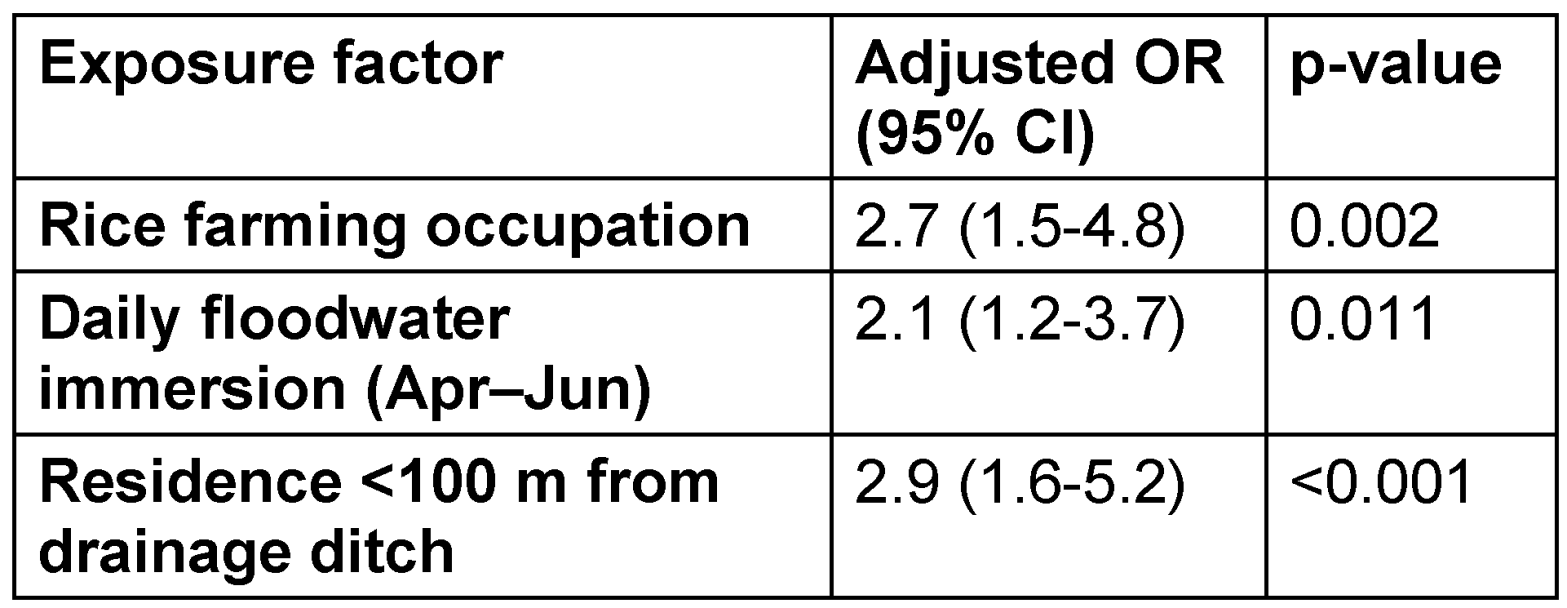
Multivariate analysis of risk factors for MAT-confirmed leptospirosis (n=41 cases, n=141 controls)
